# Relationship of physical activity to cardiovascular risk factors in an urban population of Nigerian adults

**DOI:** 10.1186/0778-7367-71-6

**Published:** 2013-04-11

**Authors:** Adewale L Oyeyemi, Olumide Adeyemi

**Affiliations:** 1 Department of Physiotherapy, College of Medical Sciences, University of Maiduguri, Maiduguri, Nigeria

**Keywords:** Health-related physical activity, Obesity, Chronic diseases, Nigeria

## Abstract

**Background:**

The burden of chronic diseases including cardiovascular disease (CVD) is increasing rapidly in Nigeria, but fewer studies have evaluated the role of physical activity in the development of CVD in this country. We examined the relationship between health enhancing physical activity and risk factors of CVD in a working population of adults in Maiduguri, Nigeria.

**Methods:**

In a cross-sectional study, we assessed health enhancing moderate-to-vigorous physical activity (MVPA) among 292 government employees (age: 20–65 years, 40% female, 24% obese and 79.8% response) using the self-administered version of International Physical Activity Questionnaire (IPAQ-SF). Time spent in walking and sitting during occupational activity was assessed as well. Anthropometric measurement of height, weight and waist circumference, and blood pressure were also measured. Independent t-test and One- Way ANOVA were conducted, and the relationships between MVPA and body mass index (BMI), waist circumference, blood pressure and heart rate were explored using Pearson correlations coefficients and multiple regression analyses.

**Results:**

The mean time spent in health enhancing MVPA (116.4 ± 101.3 min/wk) was lower than the recommended guideline of 150 min/wk sufficient for health benefits. Compared with men, more women were less physically active, obese and reported more diagnoses of component of metabolic syndrome (p < 0.05). Participants whose work activities were highly sedentary tend to accumulate less minutes of MVPA compared with those who reported their work as moderately active or highly active (p < 0.001). Health enhancing MVPA was inversely related with body mass index (BMI), waist circumference, heart rate, and systolic and diastolic blood pressure (p < 0.05).

**Conclusion:**

Physical activity level of the working population of Nigerian adults was low and was related with adverse risk factors for CVD. Promoting health enhancing physical activity at work places may be important for prevention and control of CVD among the working population in Maiduguri, Nigeria.

## Background

The burden of cardiovascular disease (CVD) and other chronic diseases is rising rapidly in Nigeria [1]. The epidemiological transition in this country has been linked to ongoing lifestyle changes of urbanization including reduced physical activity and inappropriate diets [2-4]. Urbanization in Nigeria has been characterized by increased adoption of western life styles and preference for white collar and modern jobs with attendant public health consequences [1-3]. Current estimates indicate that chronic diseases including CVD already account for 24.2% and 25.1% of all mortalities in males and females, respectively in Nigeria [1]. Nevertheless, there is clear evidence that engaging in health enhancing physical activity protects against the development of CVD including coronary heart disease and stroke, and other chronic diseases [4-8]. Health enhancing physical activity is generally considered as any activity that benefits health and functional capacity, and it includes lifestyle, fitness, recreational, occupational and sports activities rather than just exercise alone [9-12]. Engaging in any or combination of these activities regularly at moderate or vigorous intensity is an important public health recommendation, and guidelines for sufficient health-enhancing physical activity are based on moderate-to-vigorous physical activity (MVPA) [11,12].

Until very recently [13,14], most studies from Nigeria have focused on therapeutic exercise and exercise training [15-18], neglecting the health effects of everyday activities like occupational related physical activity, tasks and chores around the home and walking for different purposes that have become central to public health interventions [10,11,19,20]. Promoting multiple forms of activities that contribute to health enhancing physical activity is one of four strategies recommended by the United Nations to reduce global epidemics of chronic non-communicable diseases [21], but strategies should be evidence base. Since Nigeria is the most populated country in Africa and with attendant rising rates of chronic diseases [1,2], there is clear relevance for understanding the relationships between risk factors of CVD and health enhancing physical activity in this country.

Blood pressure and anthropometric variables like body mass index and waist circumference are good markers and predictors of CVD because they are easy to use, less expensive and have been found to be strongly and consistently associated with risk factors of CVD and other chronic diseases, including coronary heart disease, type 2- diabetes and obesity [10,22-25]. Sufficient health enhancing physical activity has been negatively associated with these markers of risk of CVD [26-31], but has been relatively unstudied in Nigeria. To our knowledge, only two studies were available from Nigeria that explored the relationship between health enhancing physical activity and risk factors of CVD [32,33]. These studies were conducted over a decade ago, one of them did not directly explore the relationship between risk factors of CVD and physical activity [33], and it is not clear if these studies can be situated within the context of present level of urbanization in Nigeria. It is important to study the working population in Nigeria because they constitute a productive segment of the Nigerian society, are the driving force of the economy and are more likely to be receptive and impacted by the ongoing lifestyle changes of urbanization. The aim of this study was to examine the relationship between health enhancing physical activity and cardiovascular disease risk factors including body mass index, waist circumference and blood pressure in a working population of adults in Maiduguri, Nigeria. A secondary objective was to investigate the effect of occupational activity on physical activity level, BMI, waist circumference and blood pressure.

## Methods

Similar to the methodology of a previous study [26], workers were recruited from work places with variations in occupational activity level (see Table 1). Six workplaces (2 each in the health sector, education sector and government ministry) with more than 100 employees were purposively recruited from federal or state government funded facilities in Maiduguri, North Eastern Nigeria. Occupational activities at the health sector typically involved meeting with patients or clients in an office setting or long-term care of patients in an institutional setting, but also included administrative and other services (e.g., kitchen workers). Work activities at the educational sector involved teaching, research and also included managerial, administrative, or clerical work. For the Government ministry, work activities descriptions were clerical, administrative or managerial in nature and involved significant sitting time on the typewriter or computer. All workers in the selected workplaces who met the eligibility criteria of being between 18 and 65 years old, not having any disability that prevents independent walking and able to complete questionnaire in English language were invited to participate in a cross-sectional study. A total of 386 workers (60 in the health sector, 63 in education sector and 255 in government ministry) were surveyed, out of whom 55 refused to participate and 23 were not present during data collection. The response rate was 79.8% overall. Of the 308 participants that completed the survey, 292 workers that comprise 102 (35.0%) women and 190 (65.0%) men, aged 44.9 ± 8.5 years provided usable physical activity data and were included in the analysis. All participants provided informed consent, and the study was approved by the Research and Ethics Committee of the University of Maiduguri Teaching Hospital, Maiduguri.

**Table 1:** Description of general health, education, and occupational characteristics of the participants

**Characteristics**	**Total (N = 292)**	**Women (n = 102)**	**Men (n = 190)**	**p-value**^**a**^
	**n (%)**	**n (%)**	**n (%)**	
Health Status				0.282
Excellent	65 (22.3)	18 (17.6)	47 (24.7)	
Good	202 (69.2)	73 (71.6)	129 (67.9)	
Fair	25 (8.5)	11 (10.8)	14 (7.4)	
Health Problem†				
Diabetes	16 (8.9)	7 (7.1)	9 (11.3)	
Heart Disease	22 (12.4)	8 (8.2)	14 (17.5)	
Hypertension	41 (23.1)	25 (25.5)	16 (20.6) ††	
High Cholesterol	12 (6.8)	9 (9.2)	3 (3.7) ††	
Metabolic Syndrome	87 (48.8)	49 (50.0)	38 (46.9) ††	
Body Mass Index				<0.001*
Normal weight	134 (45.9)	22 (21.6)	112 (58.9)	
Overweight	88 (30.1)	37 (36.3)	51 (26.8)	
Obese	70 (24.0)	43 (42.1)	27 (14.3)	
Education level				0.069
> Secondary school	256 (87.7)	88 (86.3)	168 (88.4)	
Secondary school	26 (8.9)	13 (12.7)	13 (6.8)	
< Secondary school	10 (3.4)	1 (1.0)	9 (4.7)	
Work Setting				0.051
Health sector	41 (14.1)	23 (22.5)	18 (9.5)	
Education sector	45 (15.4)	19 (18.6)	26 (13.7)	
Ministry	206 (70.5)	60 (58.8)	146 (76.8)	
Occupation activity				0.003*
Highly sedentary	62 (21.2)	33 (32.4)	29 (15.3)	
Moderately sedentary	109 (37.3)	37 (36.3)	72 (37.9)	
Moderately active	47 (16.1)	15 (14.7)	32 (16.8)	
Highly active	74 (25.4)	17 (16.7)	57 (30.0)	

BMI_ Body Mass Index.

WC_ Waist circumference.

^a^- p- value based on Chi-Square statistic;* *p < 0.05* was considered significant.

†_ Values do not add up due to missing data (13 missing data for heart disease, 6 for hypertension, and 28 for high cholesterol). ††_ Only for hypertension (p = 0.045), high cholesterol (p = 0.023) and metabolic syndrome (p = 0.048) were significant differences exist in health problems between men and women.

### Data collection

Data were collected at the workplace at a time that was convenient to both workers and the research team. Each participant completed a questionnaire to provide data regarding occupational activity, education, health status and previous diagnoses of components of metabolic syndrome. Occupational activity was defined as highly sedentary (sitting more than 75% of work time), moderately sedentary (sitting more than 50% of the work time), moderately active (moving about more than 50% of the work time), or highly active (moving about more than 75% of the work time). Educational level was classified as more than secondary education, secondary school education, and less than secondary school education. Participants’ health status was classified as excellent, good or fair. For previous diagnoses of components of metabolic syndrome, participants were to recall any diagnosis of elevated blood pressure (hypertension), elevated fasting glucose (diabetes), elevated triglycerides (hypercholesterolemia), elevated waist circumference (abdominal obesity), reduced high-density lipoprotein or heart disease by their physician or any other health workers. Participants with a known diagnosis of three or more of these conditions were considered to have the metabolic syndrome [34].

### Physical activity

The short version of the international physical activity questionnaire (IPAQ-SF) was used to assess health enhancing physical activity level of the participants. The questionnaire estimated vigorous- and moderate- intensity activities, and walking in terms of frequency (days/wk) and duration (min/day) in the last 7 days. These activity categories may be treated separately by computing the total minutes of each category in a week or multiplied by their estimated values in METs and summed to gain an overall estimate of physical activity in a week [35]. Total minute of moderate-to vigorous physical activity (MVPA) was the primary physical activity outcome in this study, and was computed by summing minutes of time per day of moderate- and vigorous-intensity activity. Meeting recommendation on physical activity for health was defined according to the global standard of accumulating at least 150 minutes of MVPA per week [11,12]. The test retest reliability (ICC = 0.33- 0.73) and concurrent validity (ρ =0.78- 0.92) of the IPAQ-SF among Nigerian adults are good and acceptable [36]. Acceptable test retest reliability (r = 0.70- 0.97) and criterion validity (r = 0.23) compared with accelerometer monitoring has also been reported for IPAQ in both the developed and developing countries [35].

### Anthropometric measures

Height (to the nearest 0.5 cm) and weight (to the nearest 0.2 kg) were measured in light clothing and without shoes, using standardized instruments. Body mass index (BMI) was calculated as body weight divided by the square of height (kg m^-2^), and participants were categorized into normal weight (18.5- 24.9 kg m^-2^), overweight (25.0- 29.9 kg m^-2^) and obese (≥ 30.0 kg m^-2^) groups according to the WHO guidelines [37]. Waist circumference measurements (to the nearest 0.5 cm) were standardized by placing the measuring tape at the level of the last rib in standing subjects [26]. The criterion for defining abdominal obesity was based on the recent international consensus cut-points of ≥ 94cm and ≥ 80cm for Sub-Saharan African men and women, respectively [34].

### Blood pressure

Resting blood pressure and heart rate were obtained using a Dinamap (model 8100/8101) digital blood pressure measuring device, after the participants have been sitting quietly for 5 minutes. Three measurements were taken at intervals of 3–5 minutes on the left arm, and the mean systolic blood pressure, diastolic blood pressure and heart rate were used in the analysis.

### Statistical analysis

Because physical activity variables are often skewed, the square root of the original variable (min/week of moderate-to-vigorous physical activity) was used in the analyses to improve normality. Raw data were used to present the descriptive statistics.

Descriptive data are presented as means and standard deviation (SD) for continuous data and proportions for categorical data. The independent t- test was conducted to compare the differences in moderate-to-vigorous physical activity (MVPA) levels and cardiovascular disease risk factors between men and women, and also to compare MVPA and risk factors between participants who reported diagnosis of components of metabolic syndrome and those not reporting. One- Way ANOVA was carried out to explore the effect of occupational activity on BMI, waist circumference, blood pressure and MVPA. Pearson correlations coefficients were computed to investigate the relationship between MVPA and each of BMI, waist circumference and blood pressure. Multiple linear regressions were conducted as a secondary analyses to model for the predictors of MVPA and to control for the confounding influence of participants’ sociodemographics on the associations between MVPA (dependent variable) and different risk factors of CVD (independent variables).

To reduce the potential for multicollinearity, only variables that seemed essential to the model were force-entered as independent variables in the first block of the regression model and removed stepwise in the second block. The modifying effects of gender on the associations between outcomes and risk factors of CVD were explored by including gender in most analyses, and variables for which there were significant interactions were separately explored for women and men. All analyses were conducted in SPSS 15.0 for Windows.

## Results

The general characteristics of the participants are shown in Table 1. More than 90% of the participants described their health as good or excellent. Hypertension (23.1%) was the most commonly reported health problem of the components of metabolic syndrome, and about half of the participants reported a previous diagnosis of metabolic syndrome (48.8%). More than 87% of the participants had more than secondary school education and most of the participants (70.5%) work in government ministry. The majority (58.5%) of the participants described their work activities as moderately or highly sedentary. About one-fourth of participants were obese (24.0%) and the prevalence of obesity was higher among women (42.1%). No participants in this study was classified as underweight (<18.5 kg m^-2^).

Table 2 shows the comparison of physical characteristics, CVD risk factors and physical activity level between men and women. Compared with men, women had significantly (p < 0.001) higher mean BMI (29.4 kg/m^2^ vs 24.8 kg/m^2^, t = 7.752) and waist circumference (96.1 cm vs 87.7 cm, t = 4.179). Notably, the mean waist circumference of women was about 16 cm greater than the established criterion for obesity and health risk in women. Compared to men (123.8 min/wk), women were less physically active accumulating only 103 min/wk of MVPA (t = −2.704, p = 0.043).

**Table 2:** Physical characteristics, physical activity level and risk factors of cardiovascular diseases overall and by gender

	**Total (n = 292)**	**Women (n = 102)**	**Men (n = 190)**	p-value*
**Variables**	**Mean ± SD**	**Mean ± SD**	**Mean ± SD**	
Age (years)	44.8 ± 8.5	44.6 ± 8.8	44.9 ± 8.3	0.697
Weight (kg)	75.3 ± 13.7	78.8 ± 14.5	73.3 ± 12.9	0.002
Height (m)	1.69 ± 0.08	1.64 ± 0.08	1.71 ± 0.08	<0.001
BMI (kg/m^2^)	26.4 ± 5.3	29.4 ± 5.6	24.8 ± 4.5	<0.001
WC (cm)	90.7 ± 16.9	96.1 ± 15.9	87.7 ± 16.7	<0.001
Heart rate (beat/min)	78.5 ± 10.9	79.2 ± 10.4	78.1 ± 11.3	0.436
SBP (mm Hg)	129.2 ± 16.8	129.4 ± 18.9	129.1 ± 15.6	0.875
DBP (mm Hg)	81.3 ± 10.5	81.5 ± 11.9	81.2 ± 9.7	0.792
MVPA (min/wk)	116.4 ± 101.3	102.7 ± 103.3	123.8 ± 99.8	0.043

SBP_ Systolic Blood Pressure.

DBP_ Diastolic Blood Pressure.

MVPA_ Moderate-to-vigorous physical activity.

*_ Based on independent t-test statistics.

Participants who reported diagnoses of metabolic syndrome compared to those not reporting were older (48.1 years vs 43.3 years, p < 0.001), have higher systolic blood pressure (140.4 mmHg vs 124.3 mmHg, p < 0.001), heart rate (80.4 beat/min vs, 77.6 beat/min, p = 0.038), waist circumference (96.9 cm vs 87.6 cm, p < 0.001) and BMI (28.8 kg/m^2^ vs 25.2 kg/m^2^, p < 0.001). Also, participants who reported diagnoses of metabolic syndrome accumulated less minutes of MVPA per week compared to participants who did not report diagnoses of metabolic syndrome (83.9 min/wk vs 132.0 min/wk, p < 0.001) (Not shown in table). A significant modifying effect of gender (p = 0.047) but not of occupation (p = 0.528) was found. While pattern of findings in men was similar to that reported in the overall sample, only systolic blood pressure (p < 0.001) and waist circumference (p = 0.003) were significant between women who reported and do not report diagnoses of metabolic syndrome. Systolic blood pressure (138.8 mmHg vs 123.3 mmHg) and waist circumference (102.8 cm vs 93.4 cm) were higher among women who reported diagnoses of metabolic syndrome than those who did not report (Not shown in table).

Self-reported occupational activity had effects on CVD risk factors including health related MVPA (Table 3). Body mass index, waist circumference and blood pressure were significantly higher among participants who reported their work activities as highly sedentary compared with participants who reported their work as moderately active or highly active (p < 0.001). On the other hands, participants who reported highly sedentary work activities tend to accumulate less minute of MVPA compared with participants who reported their work as moderately active or highly active (p < 0.001). Gender specific findings were similar to the pattern in the overall sample with Body mass index, waist circumference and blood pressure significantly higher in both men and women who engaged in highly sedentary work activities compared to their counterparts who engaged in moderately active or highly active work activities.

**Table 3:** Association between occupational activity and physical activity level, BMI, waist circumference and blood pressure

**Occupation activity (n)**	**MVPA (min/wk)**	**BMI (kg/m**^**2**^**)**	**WC (cm)**	**SBP (mmHg)**
	**Mean ± SD**	**Mean ± SD**	**Mean ± SD**	**Mean ± SD**
Highly sedentary (62)	34.1 ± 25.3	30.6 ± 5.2	104.5 ± 10.4	142.9 ± 18.7
Moderately sedentary (109)	111.7 ± 97.4	26.1 ± 4.7	89.3 ± 12.8	126.4 ± 14.6
Moderately active (47)	148.5 ± 106.6	25.7 ± 5.5	89.2 ± 23.7	125.9 ± 13.8
Highly active (74)	171.8 ± 98.1	23.7 ± 3.7	81.9 ± 14.5	123.8 ± 13.8
F- value	29.262	24.378	25.989	21.711
p- value	<0.001	<0.001	<0.001	<0.001

MVPA_ Moderate-to-vigorous physical activity.

BMI_ Body mass index.

WC_ Waist circumference.

SBP_ Systolic blood pressure.

**Figure 1:**
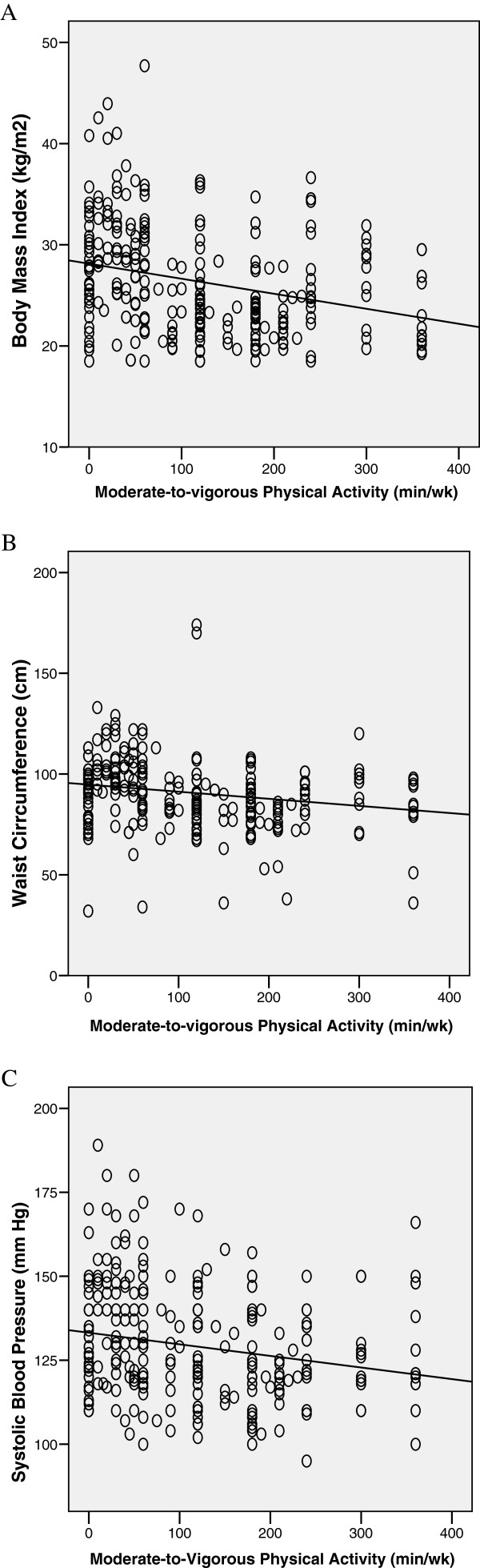
Correlation analysis of moderate-to-vigorous physical activity with A) Body mass index (r = −.32, p < 0.001), B) Waist circumference (r = −.25, p < 0.001) and C) systolic blood pressure (r = −.22, p < 0.001).

Figure 1 shows the relationships between health- related MVPA and BMI, waist circumference and systolic blood pressure. A modest inverse correlation between MVPA and BMI was detected (Figure 1A, r = − 0.319, p < 0.001). Inverse correlations were also found between MVPA and waist circumference (r = − 0.252, p < 0.001, Figure 1B), and with systolic blood pressure (r = − 0.224, p < 0.001). There was significant inverse relationship between MVPA and diastolic blood pressure (r = − 0.194, p = 0.010) but not between MVPA and heart rate (r = − 0.102, p = 0.075). Patterns of significant findings (p < 0.001) similar to the results in the overall sample were found for both men (MVPA and BMI: r = − 0.305; MVPA and WC: r = − 0.219; MVPA and SBP, r = − 0.200) and women (MVPA and BMI, r = − 0.291; MVPA and WC, r = − 0.236; MVPA and SBP: r = − 0.264).

Multiple regression analyses with age, gender, educational level, occupation and health status included as independent covariates was used to model health enhancing MVPA (Table 4). Body mass index, waist circumference and systolic blood pressure were significant predictors of health enhancing MVPA in the overall model. While similar trend of inverse significant associations for all risk factors with health enhancing physical activity in the overall model was found in men, only body mass index and systolic blood pressure were significant and inversely associated with health enhancing MVPA in women.

**Table 4:** Multiple regression models for the relationship between health enhancing moderate-to-vigorous physical activity (MVPA) and body mass index, waist circumference and blood pressure, overall and by gender

**Variables**	**Overall**		**Women**		**Men**	
	**β**	***p***	**β**	***p***	**β**	***p***
BMI	−0.297	<0.001	−0.246	0.029	−0.282	<0.001
WC	−0.185	0.005	−0.155	0.153	−0.197	0.016
SBP	−0.192	0.002	−0.187	0.037	−0.186	0.012
DBP	−0.122	0.054	0.079	0.465	−0.178	0.042
Age	0.035	0.545	−0.094	0.535	0.063	0.430
Gender	−0.005	0.935	- -	- -	- -	- -
Education	0.016	0.773	−0.009	0.927	0.010	0.884
Occupation	0.040	0.492	0.089	0.380	0.033	0.608
Health Status	−0.120	0.062	−0.056	0.655	−0.167	0.041

β- Standardized Regression Coefficient.

-- Control not made for gender because data was split by gender.

BMI- Body Mass Index, WC- Waist Circumference, SBP- Systolic Blood Pressure, DBP- Diastolic Blood Pressure.

## Discussion

This study investigated the relationship between health enhancing physical activity and risk factors of cardiovascular diseases, and explored the effect of occupational activity on BMI, waist circumference, blood pressure and health enhancing physical activity in a working population of adults in Nigeria. Health enhancing physical activity was inversely related to body mass index, waist circumference and blood pressure. Consistently, health-related physical activity level has been reported to be inversely related to risk of CVD including body mass index, waist circumference, blood pressure, high density lipoprotein (HDL) and blood cholesterol levels in occupational groups [26,32,33,35-40], and in the general population [27-30]. Our finding therefore seems to affirm a pattern that suggests that health enhancing physical activity can confer protection against the risks of CVD in a sample of Nigerian working population.

We found health enhancing physical activity to increase with more occupational activity, and more favorable CVD risk profiles among those who reported high occupational activity, suggesting that some of the relationship between health-enhancing physical activity and CVD risk factors was explained by occupational activity. Previous studies have emphasized the contribution of occupational activities to total physical activity [26,41]. Low level of occupational physical activity has also been reported to independently accentuate the risks of CVD in many population [19,39,42], including that of Nigeria [32]. Plausibly, promoting occupational based physical activity may be a viable approach for improving health enhancing physical activity and control the risk of CVD in the Nigerian working population.

Not surprising, participants whose occupational activity was highly sedentary were found to have higher BMI, waist circumference and elevated blood pressure compared to those whose work activity was not sedentary. It is possible that work places that promote sedentariness may be contributory to the risks and the development of obesity and hypertension among employees in Nigeria. This is important because obesity and hypertension are strong risk factors that could erroneously be perceived as sign of affluent and remain largely asymptomatic, respectively, until they manifest in the development of chronic diseases. Typifying this paradox is the finding that majority of the participants reported their health as good and excellent despite the fact that most of them had previous diagnoses of one or more components of metabolic syndrome, were overweight/obese and did not meet the guideline for health enhancing physical activity. It may be timely to institute public health interventions that include education on the contribution of sedentary workplaces to risk of CVD in Nigeria.

Also worth noting is the high prevalence of obesity in the present study. With both measures of adiposity (BMI and waist circumference), women were consistently more obese than men. The prevalence of obesity found was considerably higher than that of the general adult population in Nigeria (24% vs 7.8%- 13.9%) [1]. Perhaps, obesity is more of a problem to the working population in Nigeria because the influence of urbanization and adoption of western lifestyles is more pronounced among this group. However, a finding of this nature warrants urgent attention because persistent obesity can dysregulates the metabolic processes including the action of insulin on glucose-lipid-free fatty acid metabolism and can severely affects processes controlling blood glucose, blood pressure, and lipids metabolism [43,44]. This often results in a cluster of conditions including dyslipidemia, hypercholesterolaemia, hypertension, type 2- diabetes mellitus, abdominal obesity and cardiovascular diseases, known as the metabolic syndrome with dire public health consequences [34,43].

Consistent with previous studies [26,45,46], we found higher adverse risk profiles of CVD including reduced level of health enhancing physical activity in those that have been diagnosed previously with factors associated with metabolic syndrome. Evidence suggests that individuals who are less fit have a 7-fold greater chance of developing the metabolic syndrome compared to fit and physically active individuals [45], and that sedentary individuals are more susceptible to suffer from or develop the metabolic syndrome [45,46]. Regular physical activity has been recommended as an effective preventative approach to modulate the metabolic syndrome [26,43], and this may be an important public health recommendation in Nigeria where chronic disease rates are rising.

Overall, our findings underscored the potential link between health enhancing physical activity and risk factors of emergent chronic diseases of urbanization in Nigeria. Because of the tremendous socio-economic changes associated with urbanization and upsurge in rural–urban migration occurring in Nigeria, many people are likely adopting sedentary lifestyles and putting themselves at risk of chronic diseases. Motorized transportation seems to have become dominant in most urban cities in Nigeria thereby reducing active forms of transportation like walking and bicycling. This is especially apparent in the working populations who are more likely to own a car compared to the unemployed populations. Also, advances in technology and introduction of numerous modern labour saving devices into Nigeria could be contributing negatively to people’s activity patterns. Although desirable for efficiency, increasing computerization and mechanization at many government workplaces in Nigeria could lead to reduce occupational activity and subsequently to decline in overall physical activity. People in Nigeria are likely to be receptive to the advances brought by urbanization because they could be considered to be associated with improved standard of living and socioeconomic progress [2]. However, urbanization accentuates physically inactive lifestyles and increases the risk of CVD and numerous chronic diseases. Promotion of physically active lifestyles is particularly important in a developing country like Nigeria, because physical inactivity is not only the fourth leading cause of death worldwide but most of these deaths are occurring in developing countries [5,6,47].

The strength of this study was a sample that was selected to represent varying level of occupational activities that enables us to explore the independent effect of occupational activity on risk factors of CVD and health-enhancing physical activity. Also, the objective assessment of BMI, waist circumference, blood pressure and the use of current international health related guideline for sufficient health-related physical [11,12] were other strengths of this study. However, this study has some important limitations which should be considered when interpreting the findings. First, the use of government employees produced a sample of relatively high socioeconomic status which may reduce generalizability of results to other Nigerian samples, especially women and those in rural populations with traditional jobs. Our sample did not include unemployed Nigerians who were mostly women, and was limited to the urbanized population with potential for modern jobs, thereby biasing our findings in favor of employed men and urbanized population. However, previous Nigerian studies have consistently found higher prevalence of CVD risk factors and metabolic syndrome in studies of urban populations compared with rural populations [3,4,48,49], suggesting that public health burden of chronic diseases in Nigeria increases as the population transits from rural subsistence agricultural setting to a more westernized society. A second limitation was the use of self-report measure of physical activity as opposed to objective. Self-report measures of physical activity are prone to misinterpretation, social desirability bias and inaccurate recall of intensity, frequency and duration of physical activity [50,51]. Third, the study did not include measures of fasting blood glucose, serum lipid profiles and diets which are important risk factors of CVD [38,39,42]. A fourth limitation was that the cross-sectional design utilized does not allow for causal relationships to be determined. However, given the limited literature in this field in Nigeria, correlational studies such as this can provide leads for researchers and policy makers in planning future work or interventions to prevent and control the risk of CVD.

Despite these limitations, the present study of Nigerian working population may have practical relevance for policy on prevention and control of CVD in Nigeria. Generally, rapid urbanization and lifestyle changes have negatively impacted physical activity behaviors of adults in Nigeria. Particularly, the increased access to automobiles and the use of technology such as computers and elevators at work seem to be contributing to the decline in transport- and occupational- related physical activity in this country. Low occupational physical activity can obviously reduce total daily physical activity [26,41,52,53], and has been documented for many years to be consistently and independently associated with negative health status [54-56]. World-wide, the workplace is increasingly becoming the focus of detection and intervention programs that include physical activity to reduce the risk of CVD and other chronic diseases [39,40,56,57]. Given that many adults in Nigeria are employed (59% of women and 80% of men) [58], and are expected to spend a considerable part of their daily time at work, promoting and encouraging health enhancing physical activity at work places could therefore be a practical and effective approach for preventing and controlling the risk of CVD and other chronic diseases in the Nigerian population.

## Conclusions

In conclusion, low level of health-enhancing physical activity in Nigerian working population was related to elevated BMI, waist circumference and blood pressure. High occupational activity was significantly associated with more health-enhancing physical activity but with lower BMI, waist circumference and blood pressure. Engaging in health enhancing physical activity may be effective for primary prevention of CVD risk factors in Nigeria. Future studies in larger heterogeneous samples are needed in order to generalize these findings to other Nigerian population.

## Competing interests

The authors declare that there is no competing interest.

## Authors’ contributions

ALO conceived and designed the study, analyzed and interpreted the data, drafted and revised the manuscript and gave approval for the final version. OA participated in the design of the study and was responsible for data acquisition. Both authors read and approved the manuscript.
